# Facilitation of Rice Stripe Virus Accumulation in the Insect Vector by Himetobi P Virus VP1

**DOI:** 10.3390/v7031492

**Published:** 2015-03-23

**Authors:** Shuo Li, Shangshu Ge, Xi Wang, Lijuan Sun, Zewen Liu, Yijun Zhou

**Affiliations:** 1 College of Plant Protection, Nanjing Agricultural University, Nanjing 210095, China; E-Mails: lishuolstonnv989@163.com (S.L.); gss20090503@163.com (S.G.); 2 Institute of Plant Protection, Jiangsu Academy of Agricultural Sciences; Jiangsu Technical Service Center of Diagnosis and Detection for Plant Virus Diseases, Nanjing 210014, China; E-Mails: smilewangxi@163.com (X.W.); sunlj520@126.com (L.S.)

**Keywords:** rice stripe virus, Himetobi P virus, small brown planthopper, accumulation of RSV

## Abstract

The small brown planthopper (SBPH) is the main vector for rice stripe virus (RSV), which causes serious rice stripe disease in East Asia. To characterize the virus-vector interactions, the SBPH cDNA library was screened with RSV ribonucleoprotein (RNP) as bait using a GAL4-based yeast two-hybrid system. The interaction between RSV-RNP and the Himetobi P virus (HiPV, an insect picorna-like virus) VP1 protein was identified. The relationships between HiPV and RSV in SBPH were further investigated, and the results showed that the titer of RSV was commonly higher in single insect that exhibited more *VP1* expression. After the *VP1* gene was repressed by RNA silencing, the accumulation of RSV decreased significantly in the insect, whereas the virus acquisition ability of SBPH was unaffected, which suggests that HiPV VP1 potentially facilitates the accumulation of RSV in SBPH.

## 1. Introduction

Rice stripe virus (RSV) has caused serious disease in rice fields in China in the last few decades [1]. RSV is transmitted mainly by the small brown planthopper (SBPH), *Laodelphax striatellus* (Fallén), in a persistent, circulative-propagative manner [2]. The epidemic and outbreak of rice stripe disease have a close relationship with the occurrence of viruliferous SBPH populations [3]. Moreover, the latest research has shown that a few SBPHs could also transmit rice stripe disease to overseas rice fields through long-distance migration in East Asia countries [4]. Therefore, it is very crucial for disease control to research the interactions between RSV and SBPHs.

Himetobi P virus (HiPV) is an insect picorna-like virus that was originally isolated from SBPH in central Japan [5,6,7]. In the field, HiPV was only detected in SBPH populations at low incidence (0%–6%) [5]. High incidences of infection occurred in several laboratory cultures of three planthopper species: SBPH (range: 0%–100%), brown planthopper (BPH) *Nilaparvata lugens* (0%–88%), and white-backed planthopper (WBPH) *Sogatella furcifera* (0%–58%) [5]. HiPV was detected in SBPH adults and nymphs, but not in eggs, and the virus was not transovarially transmitted. HiPV infects the midgut of the host insects and is excreted into the feces, though the infected insects are asymptomatic [5,7]. The contamination from maternal insects, surrounding plant tissues and frass may be major sources of inoculum [5]. The virus is a spherical particle 29 nm in diameter, which contains a single-stranded RNA genome of molecular mass 2.8 × 10^6^ [6]. The genome of HiPV consists of 9275 nucleotides excluding the poly (A) tail, and there are two large open reading frames (ORFs) separated by a 176-nucleotide noncoding region [8]. The first ORF contains putative core motifs of picornaviral helicase, protease and RNA-dependent RNA polymerase. The coding region of three major capsid proteins VP1, VP2 and VP3 (28 kDa, 36.5 kDa and 33 kDa) is mapped onto the second ORF [8]. In the study of interaction mechanisms between RSV and SBPH, we found that RSV ribonucleoprotein (RNP) interacted with capsid protein VP1 of HiPV, which is interesting. Therefore, we investigated the relationships between HiPV and RSV in SBPH, and found HiPV potentially facilitated the accumulation of RSV in the insect vector.

## 2. Materials and Methods

### 2.1. Insects and Virus

SBPH populations used in this study were collected from Haian, Jiangsu Province, China (32.57° N, 120.45° E with an elevation of 5 m a.s.l.), and have been maintained in the laboratory for nearly ten years. High-viruliferous (RSV-infected) and RSV-free strains were screened and reared respectively in glass beakers, as described previously [9]. The monoclonal antibody and polyclonal antibody against RSV-RNP were prepared and conserved by the author’s laboratory [10].

### 2.2. Yeast Two-Hybrid Screen

A yeast two-hybrid cDNA library from high-viruliferous (RSV) SBPH populations was constructed by using a homologous recombination reaction [11]. The titer of the library was approximately 2.62 × 10^10^ cfu/mL after amplification. GAL4-based Yeastmaker™ Yeast Transformation System 2 (Clontech) was used to screen the SBPH cDNA library with RSV-RNP as the bait. The full-length sequence of the *RNP* gene was amplified from viruliferous SBPH total RNA by RT-PCR, and then cloned into the yeast expression vector pGBKT7 to produce pGBK-RNP. The bait plasmid (pGBK-RNP) and cDNA library plasmid were transformed into *Saccharomyces cerevisiae* AH109 cells using a sequential transformation protocol, and the colonies were isolated on the selective medium (SD/-Ade/-His/-Leu/-Trp plus X-α-gal) according to the user manual. Primary positive candidate plasmids containing SBPH cDNAs were isolated and then cotransformed into AH109 with the bait plasmid pGBK-RNP to repeat the two-hybrid assay. The final positive candidate plasmids were determined by sequencing analysis. The sequences of positive colonies were subsequently subjected to a BLAST search against the GenBank database.

To determine the interaction between HiPV-VP1 and RSV-RNP, the specific primers for *VP1* were designed according to the library sequences (Table 1), and full-length ORF of *VP1* was amplified by RT-PCR with RNA from viruliferous (RSV) SBPH. The *VP1* gene was cloned into the yeast expression vector to produce pGAD-VP1 and pGBK-VP1. Four sets of plasmids (pGAD-VP1/pGBK-RNP, pGAD-RNP/pGBK-VP1, positive control pGADT7-RecT/pGBKT7-53 and negative control pGADT7-RecT/pGBKT7-Lam) were cotransformed into *S. cerevisiae* AH109 cells.

### 2.3. Quantitative Detection of HiPV and RSV in a Single Insect

To investigate the relationships between HiPV and RSV in SBPH, two viral titers in a single insect were examined using real-time quantitative PCR (qRT-PCR). Total RNA from individual RSV-infected SBPH was extracted using SV total RNA isolation system (Promega) following the manufacturer’s instructions. The concentration and quality of each RNA sample were determined with a NanoDrop 2000C spectrophotometer (Thermo Scientific, Wilmington, DE, USA). The first strand cDNA was synthesized using PrimeScript™ RT Master Mix Kit (Takara, Dalian, China) according to the manufacturer’s protocols. RSV *RNP* gene and HiPV *VP1* gene were used as the target genes, and SBPH β*-actin* gene was used as an internal standard. Primers used for qRT-PCR analysis were designed using the Primer3 (http://primer3.ut.ee/) and listed in Table 1. qRT-PCR analysis was performed using an IQ5 Real-Time PCR System (Bio-Rad, Hercules, CA, USA), as described by Li *et al.* [12]. After amplification, the melting curve analysis was performed to eliminate the production of non-specific products. The C_T_ (threshold of cycle) values from all samples were obtained, and the relative expression of *RNP* and *VP1* genes in each SBPH was calculated according to ΔC_T_ algorithm [13].

### 2.4. Synthesis of dsRNA

The target sequence (nucleotide 8438–8847) of HiPV *VP1* gene was amplified by RT-PCR using HiPV cDNA and specific primers conjugated with 21 bases of the T7 RNA polymerase promoter (Table 1). The PCR product (452 bp) was used as a template for dsRNA synthesis. The green fluorescent protein (*GFP*) gene (U87973) was used as control dsRNA, and the primers T7-GFP-F and T7-GFP-R were used to amplify the *GFP* fragment (573 bp) (Table 1). The dsRNAs were prepared using the T7 Ribomax Express RNAi System (Promega). After synthesis, the dsRNA was isopropanol precipitated, resuspended in ultra-pure water, and quantified spectrophotometrically at 260 nm, and its purity and integrity were determined by agarose gel electrophoresis.

**Table 1:** PCR primers used in this study.

Primer and Purpose	Sequence (5’→3’)	Modification
**Construction for Yeast Two-Hybrid Assay**		
VP1-F	CGAATTCGCTAACTTTGCGTCTACT	EcoR I
VP1-R	AGGATCCCTAAACTTTGTCAAAGG	BamH I
RNP-F	GCATATGATGGGTACCAACAAGCC	Nde I
RNP-R	TGGATCCCTAGTCATCTGCACCTT	BamH I
**For qRT-PCR**		
qVP1-F	GGTCCAGGGTGCTTTGATTG	
qVP1-R	ACTGATGGTTGTGATGCGTG	
qRNP-F	TGCAGAAGGCAATCAATGACAT	
qRNP-R	TGTCACCACCTTTGTCCTTCAA	
qActin-F	TCTTGAGATTGGACTTGGC	
qActin-R	GTAGCACAGTTTCACCTTG	
**For dsRNA Synthesis**		
T7-VP1-F	TAATACGACTCACTATAGGGATCCTTATCCTGTGTAGGAG	
T7-VP1-R	TAATACGACTCACTATAGGGAGTTAAATATGTGGGGCAAT	
T7-GFP-F	TAATACGACTCACTATAGGGAGTGGAGAGGGTGAAGG	
T7-GFP-R	TAATACGACTCACTATAGGGAGGGCAGATTGTGTGGAC	

Underlining indicates a restriction enzyme site.

### 2.5. Feeding-Based RNA Interference (RNAi) Analysis

The dsRNA ingestion experiment was performed as described by Fu *et al.* [14] and Chen *et al.* [15], with some modifications. Briefly, the glass cylinders (12 cm in length and 2.8 cm in diameter) were used as feeding chambers for rearing SBPHs on artificial diets. Two opening ends of the chamber were enclosed with the nylon meshes and stretched Parafilm M membrane (about four times the size of the original area) after insects were introduced. The artificial diet was held between two layers of stretched Parafilm M requiring insects to puncture the inner membrane to feed, and replaced every day. The dsRNA was added to artificial diets in different doses (400 ng/μL, 150 ng/μL and 30 ng/μL) for determining effective dsRNA concentration. The chambers were covered with a piece of black cotton cloth, but the diet end was exposed to light. The rearing experiments were carried out in a growth cabinet with a humidifier at 26 °C, with a photoperiod of 16/8 h (light/dark).

### 2.6. RSV Accumulation Analysis

Viruliferous (RSV) nymphs (3rd instar) from the same strain were reared on artificial diets after being pre-starved for 3 h. Approximately 150 individuals were transferred into each chamber as repetition and four replicates were set for each RNAi treatment. After a 6-day continuous feeding, the surviving nymphs were transferred to healthy rice seedlings for one day, and then were collected for subsequent viral accumulation analysis. The expression level of HiPV *VP1* and RSV *RNP* genes in SBPH after receiving *VP1* dsRNA, *GFP* dsRNA and artificial diets without dsRNA (as negative control) was examined using qRT-PCR, according to the methods mentioned above. The other half of the insects in each treatment was used for RSV RNP protein detection. Protein extraction and Western blot were performed as described previously by Li *et al.* [9]. In the Western blot, total proteins separated by 12% SDS-PAGE were blotted onto nitrocellulose membrane (Pall, New York, USA) and probed with RNP-specific polyclonal antibody, β-actin antibody (as internal control) (Sigma-Aldrich, Saint Louis, MO, USA) and subsequent horseradish peroxidase-conjugated IgG.

### 2.7. Virus Acquisition Experiments on SBPH

RSV-free nymphs (3rd instar) were reared on artificial diets for RNAi as described above. Approximately 150 individuals were used as a repetition and at least three replicates were set for each RNAi treatment. After 6-day continuous feeding, 1/3 of the surviving nymphs in each treatment were used to examine the efficiency of RNAi by qRT-PCR. The remaining 2/3 insects were pre-starved for 3 h and transferred onto RSV-infected leaves for virus acquisition according to the method described by Li *et al.* [16]. After 24 h, SBPHs were transferred to healthy rice seedlings for a 10-day latent period and then collected for the detection of the viruliferous rate. The dot immunobinding assay (DIBA) was performed to detect RSV in a single planthopper using the RSV monoclonal antibody, as described by Wang *et al.* [10]. All data were expressed as the mean ± SE. Multiple comparisons of the means were conducted based on the Tukey’s honest significant difference (HSD) test using a statistical analysis system (SAS).

## 3. Results

### 3.1. Interactions between HiPV-VP1 and RSV-RNP

**Figure 1:**
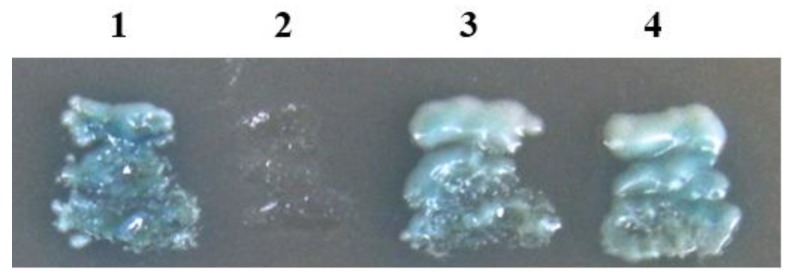
Yeast two-hybrid assay of the interaction between RSV-RNP and HiPV VP1. Yeast cotransformants were incubated on the selective medium (SD/–Ade/–His/–Leu/–Trp plus X-α-Gal) at 30 °C for 3 d. (**1**) pGADT7-RecT & pGBKT7-53 (positive control); (**2**) pGADT7-RecT & pGBKT7-Lam (negative control); (**3**) pGAD-RNP & pGBK-VP1; (**4**) pGAD-VP1 & pGBK-RNP.

To identify SBPH proteins that interacted with RSV-RNP, the SBPH cDNA library was screened using a GAL4-based yeast two-hybrid system. A number of positive colonies were obtained from approximately 2.25 × 10^8^ screened clones. Sequence analysis determined that these colonies corresponded to 12 independent proteins. One of them, with many repeated colonies, shared high amino acid sequence identity (97.3%) with capsid protein VP1 of HiPV (AB017037). Full-length ORF (774 bp) of *VP1* was cloned from SBPH and fused in-frame to the yeast expression vector. The yeast cells cotransformed with pGAD-VP1/pGBK-RNP and pGAD-RNP/pGBK-VP1 were able to grow on selective medium (SD/–Ade/–His/–Leu/–Trp supplemented with X-α-gal) (Figure 1), which confirmed the specific interactions between HiPV-VP1 and RSV-RNP in the yeast two-hybrid system.

### 3.2. Titers of HiPV and RSV in a Single SBPH

**Figure 2:**
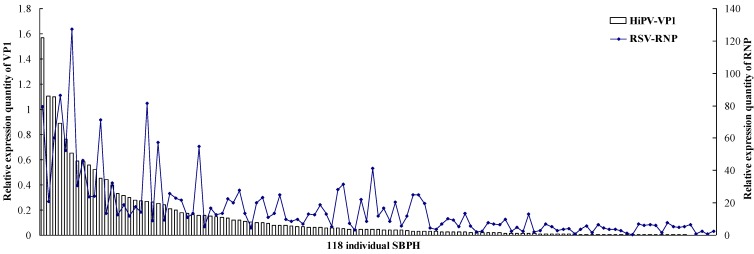
Titers of HiPV and RSV in single SBPH. After qRT-PCR, the levels of HiPV *VP1* and RSV *RNP* transcripts in single SBPH were normalized relative to the β*-actin* transcript according to the ΔC_T_ algorithm. The 118 individual SBPHs are arranged according to descending *VP1* expression quantity as the abscissa; the left ordinate indicates the expression levels of *VP1*, and the right ordinate indicates the expression levels of *RNP*.

The titers of the two viruses in a single insect were examined using qRT-PCR. As shown in Figure 2, expression levels of *VP1* and *RNP* genes were different in an individual insect, and 118 individual SBPHs were arranged according to descending *VP1* expression quantity as the abscissa. In general, the accumulation of RSV was higher in the insects that exhibited greater *VP1* expression, and when the level of *VP1* was lower, RSV titers decreased accordingly in SBPHs. In addition, the 118 individuals were divided into three groups based on expression levels of *VP1*. The first group included the individuals who expressed *VP1* above 0.1, the *VP1* relative expression of the second group is between 0.01 and 0.1, and the third group is below 0.01 (Table 2). In the first group, 89.2% of insects exhibited higher RSV accumulation, while in the third group, 63.3% of insects were lower RSV accumulation, without higher RSV accumulation insects.

**Table 2:** Classification of 118 individual SBPH based on the titers of HiPV and RSV.

Group	The Number of Insects			
	Higher RSV Accumulation	Middle RSV Accumulation	Lower RSV Accumulation	Total
First group	33 (89.2%)	3 (8.1%)	1 (2.7%)	37
Second group	23 (45.1%)	19 (37.3%)	9 (17.6%)	51
Third group	0	11 (36.7%)	19 (63.3%)	30

The 118 individuals were divided into three groups based on expression levels of *VP1*. The first group included the individuals who expressed *VP1* above 0.1, the *VP1* relative expression of the second group is between 0.01 and 0.1, and the third group is below 0.01. Higher, middle and lower RSV accumulation indicated, respectively, *RNP* relative expression above 10, 5–10, and below 5.

### 3.3. RSV Accumulation in SBPHs after Silencing of VP1

**Figure 3:**
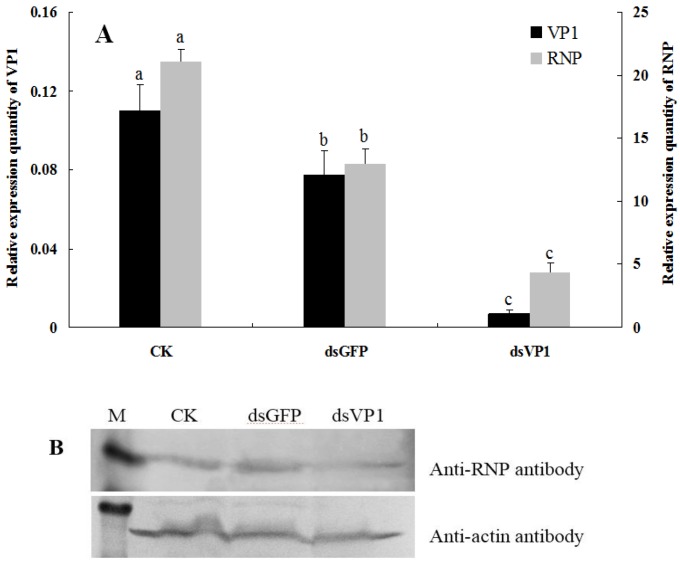
Detection of RSV accumulation levels in SBPHs after feeding-based RNA interference (RNAi). (**A**) The levels of HiPV *VP1* and RSV *RNP* transcripts in insects after feeding them artificial diets without dsRNA (CK), dsGFP and dsVP1 (with 150 ng/μl dsRNA concentration for 6 days). After qRT-PCR, the levels of *VP1* and *RNP* transcripts were normalized relative to the β*-actin* transcript according to the ΔC_T_ algorithm, and the resulting 2^−ΔCt^ values were used to plot with different feeding treatments as the abscissa. The left ordinate indicates the expression levels of *VP1*, and the right ordinate indicates the expression levels of *RNP*. Each histogram bar represents the mean (±SE) from four repeats, and the different letters above the error bars indicate significant difference as per Tukey’s honest significant difference (HSD) test (*p* < 0.05); (**B**) Detection of RSV accumulation in SBPH after different feeding treatments via Western-blot analysis. The proteins were detected using anti-RNP (above) and anti-β-actin (below) antibodies. Lane M: relative molecular weight markers.

RNAi was achieved in SBPHs by feeding them dsRNA. After 6-day continuous feeding, the survival rate of insects that fed on a high dose of dsRNA (400 ng/μL) decreased significantly, and the efficiency of RNAi with low doses of dsRNA (30 ng/μL) was also shown to be lower by detecting the transcript level of *VP1* using qRT-PCR (Supplementary Figure S1), so a medium dose of dsRNA (150 ng/μL) was used for RNAi analysis. The results showed that the abundance of *VP1* mRNA decreased significantly after feeding dsVP1 nymphs for 6 days, to approximately 6.6% of negative control and 9.3% of dsGFP control (Figure 3A), indicating that the *VP1* gene is susceptible to RNAi silencing using the feeding method. Meanwhile, *RNP* mRNA knockdown was also found in feeding dsVP1 nymphs, a 79.2% and 66.3% decrease when compared to the negative control and dsGFP control, respectively (Figure 3A). To confirm the knockdown of RSV RNP at the protein level, RNP was probed from insect proteins from three treatments via Western blot. As shown in Figure 3B, the accumulation quantity of RNP in dsVP1-feeding nymphs was lower than that in the negative control and dsGFP control nymphs. This result suggested that when the expression of HiPV VP1 was repressed, the processes of RSV replication and accumulation also slowed down in SBPHs.

### 3.4. Virus-Acquisition Ability of SBPHs after RNAi

**Figure 4:**
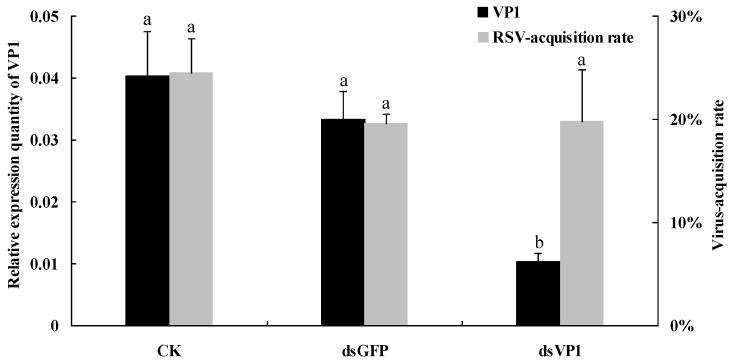
RSV-acquisition ability of SBPH after feeding-based RNAi. After qRT-PCR, the levels of *VP1* transcripts in SBPH via different feeding treatments (CK, dsGFP and dsVP1; 150 ng/μL dsRNA concentration for 6 days) were normalized relative to the β*-actin* transcript according to the ΔC_T_ algorithm. The virus-acquisition rate of SBPH was detected using the DIBA method after a 10-day latent period. The left ordinate indicates the expression levels of *VP1*, and the right ordinate indicates RSV-acquisition rate of SBPH. Each histogram bar represents the mean (±SE) from three (CK and dsGFP) or five (dsVP1) repeats, and the different letters above the error bars indicate significant difference as per Tukey’s honest significant difference (HSD) test (*p* < 0.05).

After 6-day continuous feeding, RSV-free nymphs were transferred onto RSV-infected leaves for virus acquisition. The examination of silencing efficiency indicated that *VP1* mRNA was a significant knockdown in dsVP1-feeding nymphs, with a 25.6% and 31.0% level of negative control and dsGFP control, respectively (Figure 4). The viruliferous rate of SBPH was determined after a 10-day latent period. The results showed that the virus-acquisition rates of SBPH from different treatments were similar without a significant difference (Figure 4), suggesting that the expression of VP1 did not affect RSV-acquisition ability of SBPH.

## 4. Discussion

In recent years, virus-vector-plant relationships, particularly the interactions between viruses and insect vectors, have received increasing attention. During the last few decades, RSV has caused serious disease outbreaks in rice fields in China [1]. Understanding the interactions between RSV and SBPHs can help to forecast, control and manage rice stripe disease. In this paper, to characterize the virus-vector interactions, the specific interaction factors of RSV-RNP in SBPH were investigated using a GAL4-based yeast two-hybrid screen, where we identified a series of positive colonies, including an unexpected HiPV VP1. In addition, all planthopper hosts of HiPV are important insect vectors for rice viruses; the SBPH is the vector for RSV and rice black-streaked dwarf virus [2], and BPH transmits rice ragged stunt virus and rice grassy stunt virus [2] while WBPH transmits southern rice black-streaked dwarf virus [17]. All of these viruses propagate in their vectors, so it is very significant to investigate whether HiPV affects the replication and transmission of any of these important viruses. To this end, we further analyzed the relationships between HiPV and RSV in SBPH. Quantitative analysis of the two viruses in a single insect indicated that when the level of *VP1* was higher in SBPH, there were also higher quantities of RSV.

Due to HiPV not being transovarially (vertically) transmitted, and the fact that contamination from maternal insects, surrounding plant tissues and frass may be major sources of inoculum [5], it is difficult to screen HiPV-free SBPH populations. To further analyze the effects of HiPV on the accumulation and transmission of RSV in SBPH, the feeding-based RNAi was used to silence the expression of the HiPV *VP1* gene for depressing levels of HiPV. The dsRNA-mediated RNAi has emerged as one of the most promising tools to study gene function, particularly in organisms for which stable transgenesis is not available, such as insects [18]. Compared with microinjection, though dsRNA feeding makes it difficult to monitor the precise amount of dsRNA uptaken by the insect, it is thought to be a noninvasive, simplified and promising alternative for insect RNAi [18,19]. Previous studies have demonstrated that feeding-based RNAi can successfully lead to gene silencing in several insects [19,20,21], especially in the Hemiptera species BPH [14]. Here, we found that dsRNA feeding can also specifically lead to gene silencing in the SBPH. RNAi experiments showed that after silencing the HiPV capsid protein *VP1* gene, the accumulation quantity of RSV RNP decreased significantly in SBPHs, whereas the virus-acquisition ability of SBPHs was unaffected, which suggests that HiPV VP1 potentially facilitates the accumulation of RSV in insect vector.

During the long-term evolutionary process, virus interactions with insect vectors became complex and diverse [22], and the parasitic (symbiotic) microorganisms in insects might influence virus transmission, such as chaperon GroEL and *Wolbachia*. The chaperon protein GroEL (63 kDa) is secreted by bacterial endosymbionts, and potentially plays a crucial role in virus (*Luteovirus*, *Begomovirus*, *etc.*) transmission in aphids and whiteflies by binding to virus particles and protecting them from rapid proteolytic degradation in the gut and haemolymph [23,24,25]. The endosymbiotic *Wolbachia* is an obligatory, intracellular, gram-negative bacterium that infects a wide range of arthropods and nematodes [26]. *Wolbachia* inhibits viral replication and dissemination in the main Dengue virus vector, *Aedes aegypti* [27,28]. So far, there is still little research about symbiotic microorganisms in SBPHs and their contribution to RSV transmission. The interaction between GroEL and Tenuivirus has not been reported. In our previous study, a virus overlay assay of protein blots was used to investigate the RSV-vector interactions, and binding between GroEL and RSV RNP was not found [9]. None of the virus-interacting proteins obtained via the yeast two-hybrid screen in this study were GroEL. In addition, Zhang *et al.* [29] showed the number of transcripts of *Wolbachia* in RSV-free SBPHs was four times higher than that of viruliferous (RSV) insects, but it is not clear whether this expression bias is associated with RSV infection.

In this study, we first reported that the parasitic virus HiPV contributed to RSV propagation in SBPHs. Two potential mechanisms were considered to be responsible for this case. One reason might be for the protection of HiPV. HiPV is an obligate parasite with an asymptomatic and innocuous infection in the SBPH [5], although RNAi defenses against HiPV were observed in the insect [30], HiPV should be able to better adapt and suppress the defense responses of the host. Therefore, when the level of HiPV was higher, the defense responses of SBPH were inhibited, and to a certain extent, RSV was protected from insect’s immune attack. Whether or not HiPV VP1 exhibits gene silencing suppressor activity remain to be further elucidated. The second reason involves the efficiency of viral mRNA translation. The specific interaction between VP1 and the eukaryotic initiation factor 2 (eIF2) of SBPH were identified via a yeast two-hybrid screen of cDNA library (data not shown). During the translocation step of the translation reaction, the transformation of ribosome from the pre-translocational state to the post-translocational state leads to the A- and P-site bound tRNAs moving to the P and E sites, respectively [31]. The translocation step is catalyzed by eEF2 in eukaryotes (similar to elongation factor EF-G in prokaryotes), in which eEF2 acts via a GTPase switch mechanism [32,33]. The eEF2 binds to the eukaryotic 80S ribosome via a large conformational change to facilitate tRNA translocation [34]. It was hypothesized that the interaction between VP1 and eEF2 might favor conformational change or stability of eEF2 upon binding to the 80S ribosome, which potentially improved the efficiency of RSV protein synthesis. The true biological mechanisms by which HiPV VP1 facilitates the accumulation of RSV in SBPH remained to be elucidated.

At present, the transovarial transmission mechanism of RSV by SBPH has been revealed [35], whereas the horizontal transmission mechanisms of RSV remain unclear. In general, in the process by which the insect acquires the circulative virus, the virus must cross the midgut epithelial barrier through a receptor-mediated transcytosis mechanism where the vector’s receptor proteins interact with virions or viral proteins [22,36,37,38,39]. The midgut epithelial receptor determines virus acquisition of the vector. Although HiPV infects primarily the midgut of the insect [7], our results showed that RSV-acquisition ability of the SBPH was not affected by HiPV VP1; therefore, VP1 is obviously not an epithelial receptor of RSV in the SBPH. So far, the epithelial receptor of RSV in vector midgut has not been identified, and the other 11 virus-interacting proteins obtained via the yeast two-hybrid screen in this study are not membrane proteins, so they also not potential receptors involved in the transcytosis. It is crucial to crack RSV transmission mechanisms for the SBPH to identify virus-specific receptors, which should receive more attention.

## Supplementary Files

Supplementary File 1
