# Supplementary material for: Facilitation of Rice Stripe Virus Accumulation in the Insect Vector by Himetobi P Virus VP1
Source: Viruses. 2015 Mar 23;7(3):1492–504. doi: 10.3390/v7031492 (PMC4379582; doi:10.3390/v7031492)
Supplement: Supplementary File 1 [file viruses-07-01492-s001.pdf]

Supplementary Files

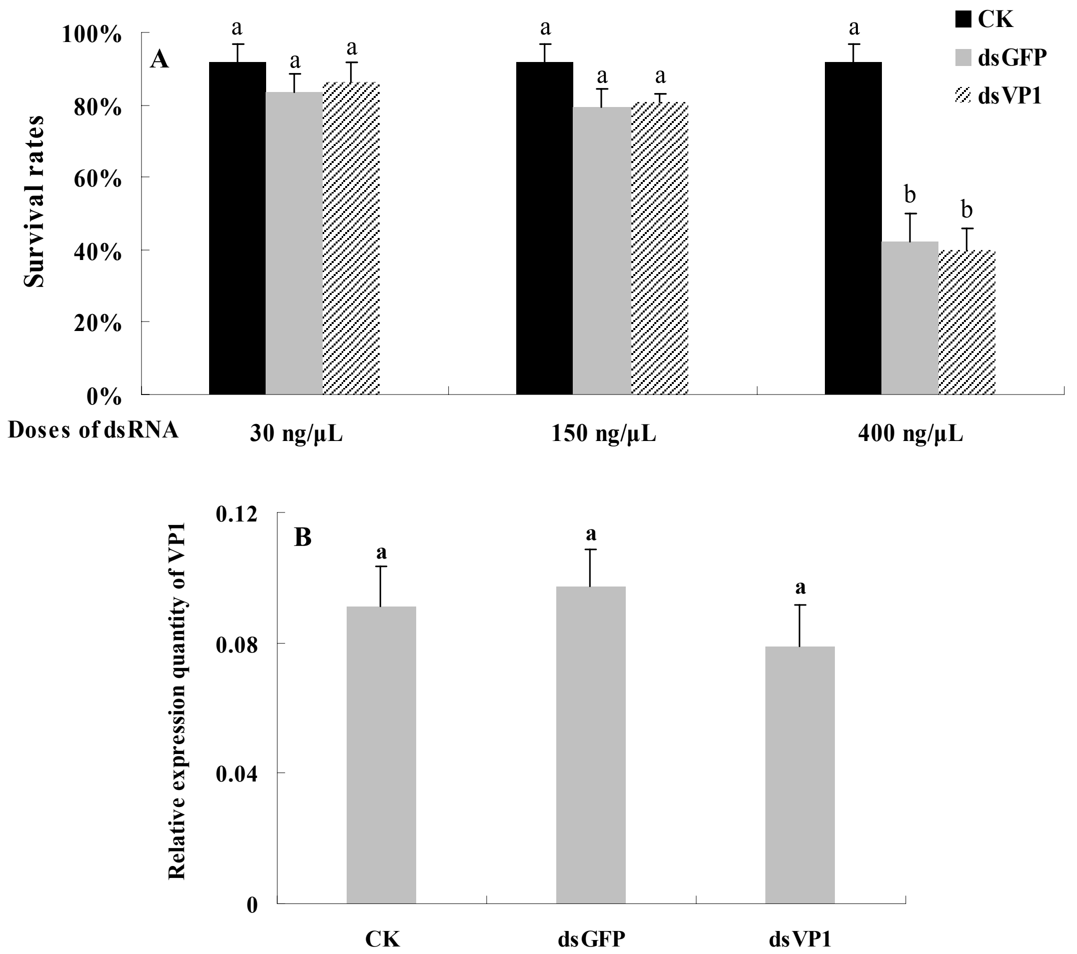

Figure S1. The survival rate and RNAi efficiency of SBPH in the dsRNA ingestion experiment. (**A**) The survival rate of SBPH after feeding different dose dsRNA for 6 days. CK is feeding the artificial diets without dsRNA. (**B**) The efficiency of RNAi in SBPH after feeding low doses of dsRNA (30 ng/μL). After qRT-PCR, the levels of HiPV *VP1* transcripts were normalized relative to the β-*actin* transcript according to the ΔC_T algorithm. Each histogram bar represents the mean (±SE) from three repeats, and the different letters above the error bars indicate significant difference as per Tukey's honest significant difference (HSD) test ($p < 0.05$).
